# In-frame seven amino-acid duplication in *AIP* arose over the last 3000 years, disrupts protein interaction and stability and is associated with gigantism

**DOI:** 10.1530/EJE-17-0293

**Published:** 2017-06-19

**Authors:** Roberto Salvatori, Serban Radian, Yoan Diekmann, Donato Iacovazzo, Alessia David, Plamena Gabrovska, Giorgia Grassi, Anna-Marie Bussell, Karen Stals, Astrid Weber, Richard Quinton, Elizabeth C Crowne, Valentina Corazzini, Lou Metherell, Tara Kearney, Daniel Du Plessis, Ajay Kumar Sinha, Atik Baborie, Anne-Lise Lecoq, Philippe Chanson, Olaf Ansorge, Sian Ellard, Peter J Trainer, David Balding, Mark G Thomas, Márta Korbonits

**Affiliations:** 1 Johns Hopkins University School of MedicineBaltimore, Maryland, USA; 2 William Harvey Research InstituteBarts and the London School of Medicine, Queen Mary University of London, London, UK; 3 Department of EndocrinologyC.I. Parhon National Institute of Endocrinology, ‘C. Davila’ University of Medicine and Pharmacy, Bucharest, Romania; 4 Research Department of GeneticsEvolution and Environment, University College London, London, UK; 5 Centre of Bioinformatics and System BiologyDepartment of Life Sciences, Imperial College London, London, UK; 6 Department of Molecular GeneticsRoyal Devon and Exeter Foundation Trust, Exeter, UK; 7 Department of Clinical GeneticsLiverpool Women’s Hospital, Liverpool, UK; 8 Department of EndocrinologyNewcastle-upon-Tyne Hospitals & Institute of Genetic Medicine, Newcastle University, Newcastle, UK; 9 Bristol Royal Hospital for ChildrenUniversity Hospitals Bristol Foundation Trust, Bristol, UK; 10 Endocrinology and Neuropathology UnitSalford Royal Hospital, Manchester, UK; 11 The Walton Centre for Neurology and NeurosurgeryLiverpool, UK; 12 Assistance Publique-Hôpitaux de ParisHôpital de Bicêtre, Service d’Endocrinologie et des Maladies de la Reproduction and Centre de Référence des Maladies Endocriniennes Rares de la Croissance, Le Kremlin-Bicêtre, France; 13 Inserm 1185Fac Med Paris Sud, Univ Paris-Sud, Université Paris-Saclay, Le Kremlin-Bicêtre, France; 14 NeuropathologyUniversity of Oxford, Oxford, UK; 15 Institute of Biomedical and Clinical ScienceUniversity of Exeter Medical School, Exeter, UK; 16 Department of EndocrinologyChristie Hospital, Manchester, UK; 17 Centre for Systems GenomicsSchools of Biosciences and of Mathematics & Statistics, University of Melbourne, Melbourne, Australia

## Abstract

**Objective:**

Mutations in the aryl hydrocarbon receptor-interacting protein (*AIP*) gene are associated with pituitary adenoma, acromegaly and gigantism. Identical alleles in unrelated pedigrees could be inherited from a common ancestor or result from recurrent mutation events.

**Design and methods:**

Observational, inferential and experimental study, including: *AIP* mutation testing; reconstruction of 14 *AIP*-region (8.3 Mbp) haplotypes; coalescent-based approximate Bayesian estimation of the time to most recent common ancestor (tMRCA) of the derived allele; forward population simulations to estimate current number of allele carriers; proposal of mutation mechanism; protein structure predictions; co-immunoprecipitation and cycloheximide chase experiments.

**Results:**

Nine European-origin, unrelated c.805_825dup-positive pedigrees (four familial, five sporadic from the UK, USA and France) included 16 affected (nine gigantism/four acromegaly/two non-functioning pituitary adenoma patients and one prospectively diagnosed acromegaly patient) and nine unaffected carriers. All pedigrees shared a 2.79 Mbp haploblock around *AIP* with additional haploblocks privately shared between subsets of the pedigrees, indicating the existence of an evolutionarily recent common ancestor, the ‘English founder’, with an estimated median tMRCA of 47 generations (corresponding to 1175 years) with a confidence interval (9–113 generations, equivalent to 225–2825 years). The mutation occurred in a small tandem repeat region predisposed to slipped strand mispairing. The resulting seven amino-acid duplication disrupts interaction with HSP90 and leads to a marked reduction in protein stability.

**Conclusions:**

The c.805_825dup allele, originating from a common ancestor, associates with a severe clinical phenotype and a high frequency of gigantism. The mutation is likely to be the result of slipped strand mispairing and affects protein–protein interactions and AIP protein stability.

## Introduction

Although the majority of pituitary adenomas are sporadic, they co-occur in families in about 2–5% of the cases (1). Rarely, they form a part of multi-glandular tumor syndromes, such as MEN1 or Carney complex. More frequently, these families present with isolated pituitary adenomas (familial isolated pituitary adenoma (FIPA) syndrome). A significant proportion (~20%) of these pedigrees carry one of a variety of heterozygous alleles in the aryl hydrocarbon receptor-interacting protein gene (*AIP*, MIM: 60555) (2, 3). AIP is a 330 amino-acid protein comprised of an N-terminal immunophilin-like domain (4) and a C-terminal domain with three tetratricopeptide (TPR) repeats (5) that are thought to be responsible for protein–protein interactions (1, 6). Disease-associated *AIP* alleles reported to date are mainly the result of nonsense, missense, deletion/insertion or splice site mutations, but a few large deletions and a promoter mutation have also been described (1, 7). *AIP* acts as a tumor suppressor gene, requiring a second somatic hit affecting the wild-type (WT) allele, with loss of heterozygosity identified in a number of cases (6, 8, 9).

Mutations leading to a truncated protein are dispersed over the entire gene, while missense mutations are more common in the C-terminal end of the molecule (6). Several alleles have been reported in different geographic regions and populations, raising the question whether they arose through recurrent mutation events (occurring at mutational hotspots in the gene) or are inherited from a recent common ancestor. The c.910C > T, p.R304* allele, for example, has been identified in several populations independently (Irish, Romanian, English, Italian, Indian, Mexican) (10, 11), consistent with this CpG locus being a mutational hotspot, but in two instances, it has also been shown to give rise locally to numerous patients originating from the same ancestor (Ireland and Italy) (10, 12).

We have previously reported the c.805_825dup, p.F269_H275dup *AIP* exon 6 in-frame duplication (rs267606578/EF643650) in three affected members of a family from the United Kingdom (3) and in an apparently sporadic giant from France (13). Here, we report seven additional pedigrees (four from the United Kingdom and three from the USA) with the same allele and provide evidence that they are all derived from a common ancestor. Using a coalescent-based approximate Bayesian computation approach, we estimate the time to most recent common ancestor (tMRCA), and by forward simulation, we estimate the current number of carriers. We hypothesize that slipped strand mispairing led to this unusual duplication and the inserted amino-acids render the protein extremely unstable and, at the same time, disrupt the binding site for crucial partner proteins.

## Patients and methods

**Figure 1:**
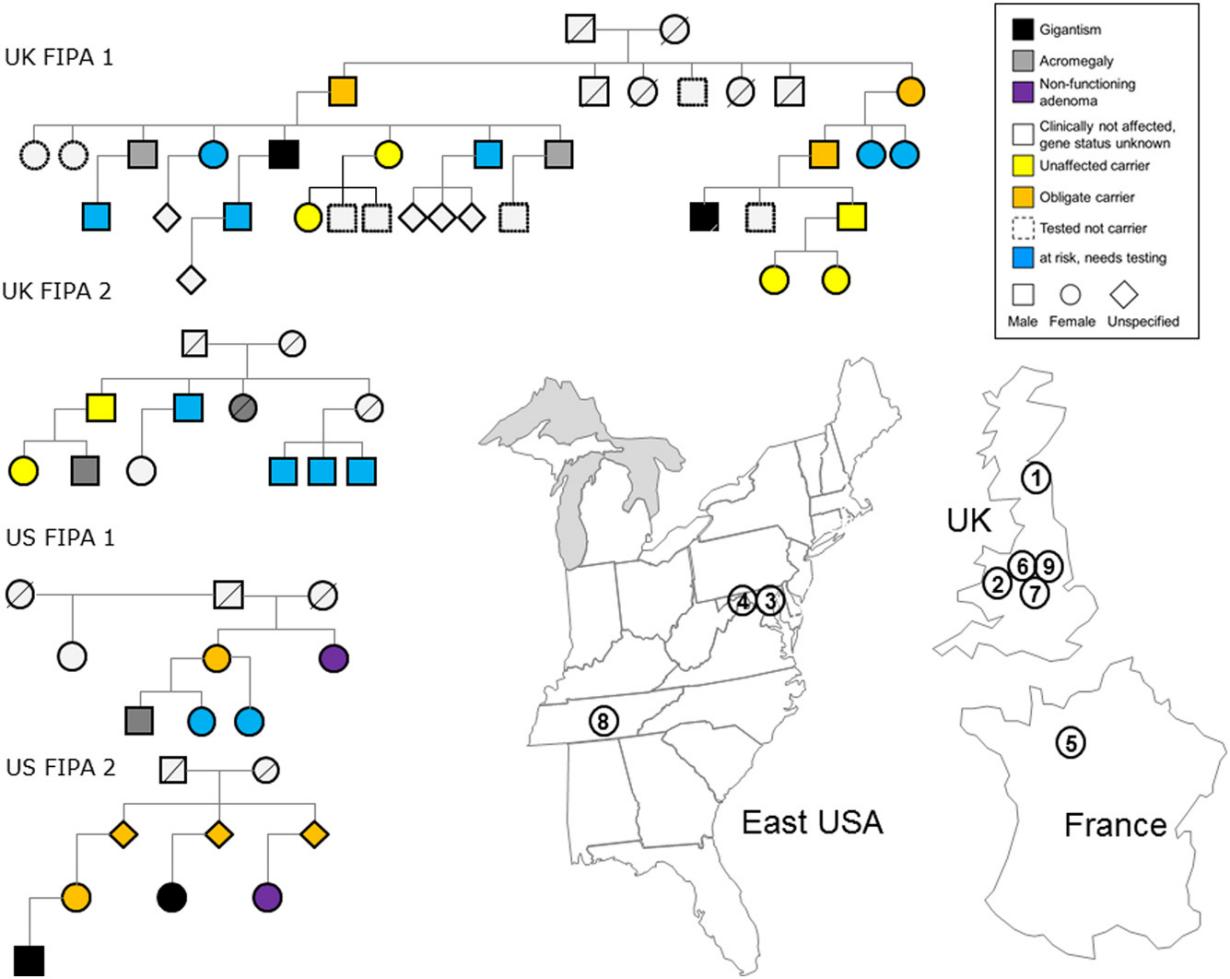
Pedigrees of the FIPA families harboring the c.805_825dup, p.F269_H275dup *AIP* mutation and map of geographical locations of the *AIP* c.805_825dup pedigrees. Pedigree numbers correspond to the first column in Table 1. A full colour version of this figure is available at http://dx.doi.org/10.1530/EJE-17-0293.

The study protocol was approved by the local ethics committees. All participants provided signed informed consent before the study. *AIP* genotyping (sequencing and multiplex ligation-dependent probe amplification) was performed as previously published (9, 14). We report nine c.805_825dup-positive pedigrees (Table 1, Fig. 1): two previously described, diagnosed in the United Kingdom (3) and France (13), and seven new ones, four diagnosed in the United Kingdom and three in the USA. All individuals have primarily European ancestry, with a known family link to the United Kingdom, but were not known to be related; they were living in France, different United Kingdom counties and two USA states (Maryland and Tennessee) (Fig. 1). Four of the nine pedigrees were FIPA families (Fig. 1), while five patients presented as simplex (apparently sporadic) cases.

**Table 1:** Clinical features of the patients carrying the *AIP* c.805_825dup, p.F269_H275dup mutation.

No	Pedigree ID	**Pituitary adenoma phenotype**	**No. of affected individuals**	**Affected individuals details** (sex, age at onset/diagnosis – years, tumor size, diagnosis)	**Treatment for pituitary disease**	**No. of unaffected carriers**	**Unaffected carriers details** (gender, age at evaluation – years)
1	UK FIPA 1 (3, 27)	GH	4	M, 11/15, Hyperplasia, Gigantism	TSS	5	F, 6; F, 6; F, 34; M, 34; F, 58
				M, 15/26, Macro, Gigantism	TSS, SSA, DA		
				M, 27.5/29, Macro, Acromegaly	TCS		
				M, na/47, Micro, Acromegaly*,GIST	TSS		
2	UK FIPA 2	GH	2	M, 22/23, Macro, Gigantism	TSS, XRT	2	F, 32; M, 71
				F, 24/28, nk, Acromegaly	TSS		
3	US FIPA 1	GH/NFPA	2	F, 22/23, Macro, NFPA (silent GH pos)	TSS, XRT	1	F, na, 55 (obligate carrier)
				M, 25/26, Macro, Acromegaly (apoplexy)	TSS 2x, XRT, SSA, pegvisomant		
4	US FIPA 2	GH/NFPA	3	M, 17/17, Macro, Gigantism	TSS	1	F, na, 60 (obligate carrier)
				F, 12/13, Macro, Gigantism	TCS + XRT		
				F, 34/40, Macro, NFPA (immunostaining not available)	TSS		
5	FranceSimplex (13)	GH	1	M, 9/14, Macro, Gigantism, psychosis	TSS+XRT+SSA+DA	–	–
6	UK Simplex 1	GH	1	M, 20/21, Macro, Gigantism	TSS 2x, SSA, pegvisomant	–	–
7	UK Simplex 2	GH	1	M, 13/18, Macro, Gigantism	TSS, XRT	–	–
8	US Simplex	GH	1	M, 17/17, Macro, Gigantism	TSS, SSA, XRT, pegvisomant	–	–
9	UK Simplex 3	GH	1	M, 18/23, Macro, Acromegaly	TCS, XRT	1	F, 66

Definition of gigantism: abnormally high growth speed in children or teenagers with abnormal IGF-1 and GH during OGTT and/or height >3 s.d. above the mean height for age or >2 s.d. over the calculated midparental height.

* Asymptomatic patient prospectively diagnosed with acromegaly following genetic testing, TSS after 3 years of medical therapy. Also has GIST tumor under surveillance.

DA, dopaminergic therapy; GIST, gastrointestinal stromal tumor; na, not applicable; nk, not known; SSA, somatostatin analogue therapy, TCS, transcranial surgery; TSS, trans-sphenoidal surgery; XRT, radiation therapy.

**Figure 2:**
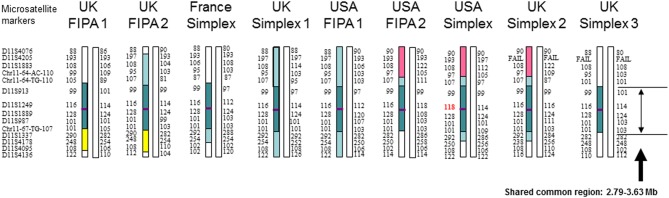
Haplotype analysis of the nine *AIP* c.805_825dup pedigrees. Colored chromosomes carry the *AIP* mutation (purple stripe): dark shading indicates the conserved haploblock shared between all mutated chromosomes; light shading shows portions of the haplotype extending to the entire genotyped region in UK Simplex 1/USA FIPA 1 pedigrees; yellow/pink shading shows additional ‘private’ sharing between closely related chromosomes. An allele mutation occurred at D11S1249 in patient US Simplex 2 (red type font). The order of individuals is arbitrary to highlight the extended haplotype sharing between pedigrees. Pedigree codes correspond to Table 1. A full colour version of this figure is available at http://dx.doi.org/10.1530/EJE-17-0293.

We genotyped 14 short tandem repeat loci (microsatellites) around *AIP* (Fig. 2) in the index case of each pedigree, and in a second individual in each of the United Kingdom FIPA 1 and 2 pedigrees, as previously described (10). Haplotypes were inferred using PHASE (15), incorporating phasing information from UK FIPA 1 and 2 pedigrees. To obtain tMRCA estimates, we used a combined approach of coalescent theory calculations and simulation in an approximate Bayesian computation framework (16). We used *ms* (17) for the simulations, combining recombination and mutation rates in order to assess the probability of neither event in the region of interest, as previously described (11). Based on the tMRCA estimates, we employed a forward simulation approach (10, 11) to estimate the current number of carriers per generation. We reject simulations that generated <9 carriers, because we observed in our dataset at least 9 unrelated carriers.

A structural model for the mutated C-terminal domain of the AIP protein was generated by template-based modeling, using the Phyre2 prediction server (18) (top templates PDB: 4aif and 4gcn; Phyre2 confidence score 100.0% and 99.6% respectively). Phyre2 uses the alignment of Hidden Markov models via HHsearch (19) to significantly improve the accuracy of alignment and detection rate. A match with a confidence score >90% indicates that the core of the protein is modeled at high accuracy (2–4 Å root mean square deviation from the native, true structure). However, surface loops can deviate from the native. Regions not covered by templates are modeled *ab initio* using the Poing algorithm embedded in Phyre2 (18).

The interaction between mutant AIP and HSP90, a well-established AIP-interacting partner (5), was investigated by co-immunoprecipitation. The coding sequence of *AIP* with the c.805_825dup, p.F269_H275dup mutation (20) was sub-cloned into the pcDNA3.0 vector C-terminally to the Myc tag epitope (3). Myc-tagged p.F269_H275dup AIP and HA-tagged HSP90β were co-transfected in HEK293 cells (10 × 10^6^, 20 µg of total plasmid DNA), using Lipofectamine 2000 (Life Technologies) following the manufacturer’s instructions. Cells were harvested 48 h later by trypsinization and resuspended in 1.5 mL of lysis buffer (150 mM NaCl, 10 mM Tris–HCl pH 7.5, 10% v/v glycerol, 1% v/v IGEPAL CA-630 (Sigma-Aldrich) supplemented with Complete Protease Inhibitor Cocktail (Roche)). After being cleared by centrifugation, lysates were cleaned up by incubation with 50 µL of Protein G Sepharose 4 Fast Flow (GE Healthcare) and divided in thirds for incubation with 5 µg of anti-Myc (Santa Cruz Biotechnology; clone 9E10) or anti-HA (Sigma-Aldrich H3663) mouse monoclonal antibodies, or mouse IgG (Sigma-Aldrich I5381), as appropriate. The co-immunoprecipitation was performed as previously described (21), and the eluates were resolved by denaturing polyacrylamide gel electrophoresis followed by Western blot using anti-Myc (Santa Cruz Biotechnology; clone 9E10) and anti-HA (Sigma-Aldrich H6908) antibodies. The experiment was repeated twice for confirmation.

Protein stability was assessed by cycloheximide chase experiments, as previously described (21). Briefly, Myc-tagged WT, p.F269_H275dup and p.R304* AIP were overexpressed in HEK293 cells, and the cells were subsequently treated with cycloheximide 20 μg/mL (Abcam) for the time indicated. Cell lysates were prepared for polyacrylamide gel electrophoresis and Western blot with anti-Myc (Santa Cruz Biotechnology; clone 9E10) and anti-GAPDH (Santa Cruz Biotechnology FL-335) antibodies. The normalized protein levels were expressed as percentage of those observed at time 0. The results of at least two independent experiments, in triplicates, were pooled together and analyzed using a one-phase decay equation. The degradation constant (K) was compared between the mutants and the WT protein using the extra sum-of-squares *F* test. Significance was set for *P* values <0.05.

### Ethical approval

All procedures performed in studies involving human participants were in accordance with the ethical standards of the institutional and national research committee and with the 1964 Helsinki declaration and its later amendments or comparable ethical standards.

## Results

The majority of the c.805_825dup-positive affected individuals presented with somatotropinoma (14 of 16 patients), but two clinically non-functioning pituitary adenomas (NFPA) were also observed (Table 1). Two FIPA families were homogenous, with all affected members harboring somatotropinomas and the other two were heterogeneous with both somatotropinomas and clinically NFPAs (Fig. 1). From a total of 16 affected allele carriers (4F/12M), 13 patients (10M) presented with signs/symptoms of pituitary macroadenoma: eight gigantism (1F/7M), three acromegaly (3M), 2 clinically non-functioning pituitary adenomas (2F, one with 5–10% GH-positive cells while for the other immunostaining is not available) (Table 1). One asymptomatic male patient (UK FIPA 1), identified prospectively after genetic screening, had a GH-secreting pituitary microadenoma (IGF-1 = 1.5 × upper limit of normal) and a 1 cm gastrointestinal stromal tumor; after three years of medical therapy, his pituitary adenoma was successfully operated on, while he remains under surveillance for the gastrointestinal stromal tumor. One patient, originally operated at the age of 15 years when histology showed somatotroph hyperplasia, was suffering from diabetes and hypertension and died following leg surgery at the age of 35 years (UK FIPA 1). One subject (France Simplex) affected by gigantism had dilated cardiomyopathy, aortic dilatation and history of infantile psychosis.

The overall average age of onset of symptoms was 19.1 years (median 18.5, range 9–34).

Haplotype analysis showed a 2.79–3.63 Mbp (1.83–2.59 cM) haploblock around *AIP* shared among c.805_825dup allele-carrying chromosomes in all nine pedigrees, as well as additional haploblocks shared among subsets of chromosomes, strongly supporting the existence of a recent common ancestor; the ‘English founder’ (Fig. 2). We observed a different allele of D11S1249 in one subject (US Simplex), on the c.805_825dup allele-carrying haplotype and conditioned our coalescent simulation results on the occurrence of a mutation at this locus.

**Figure 3:**
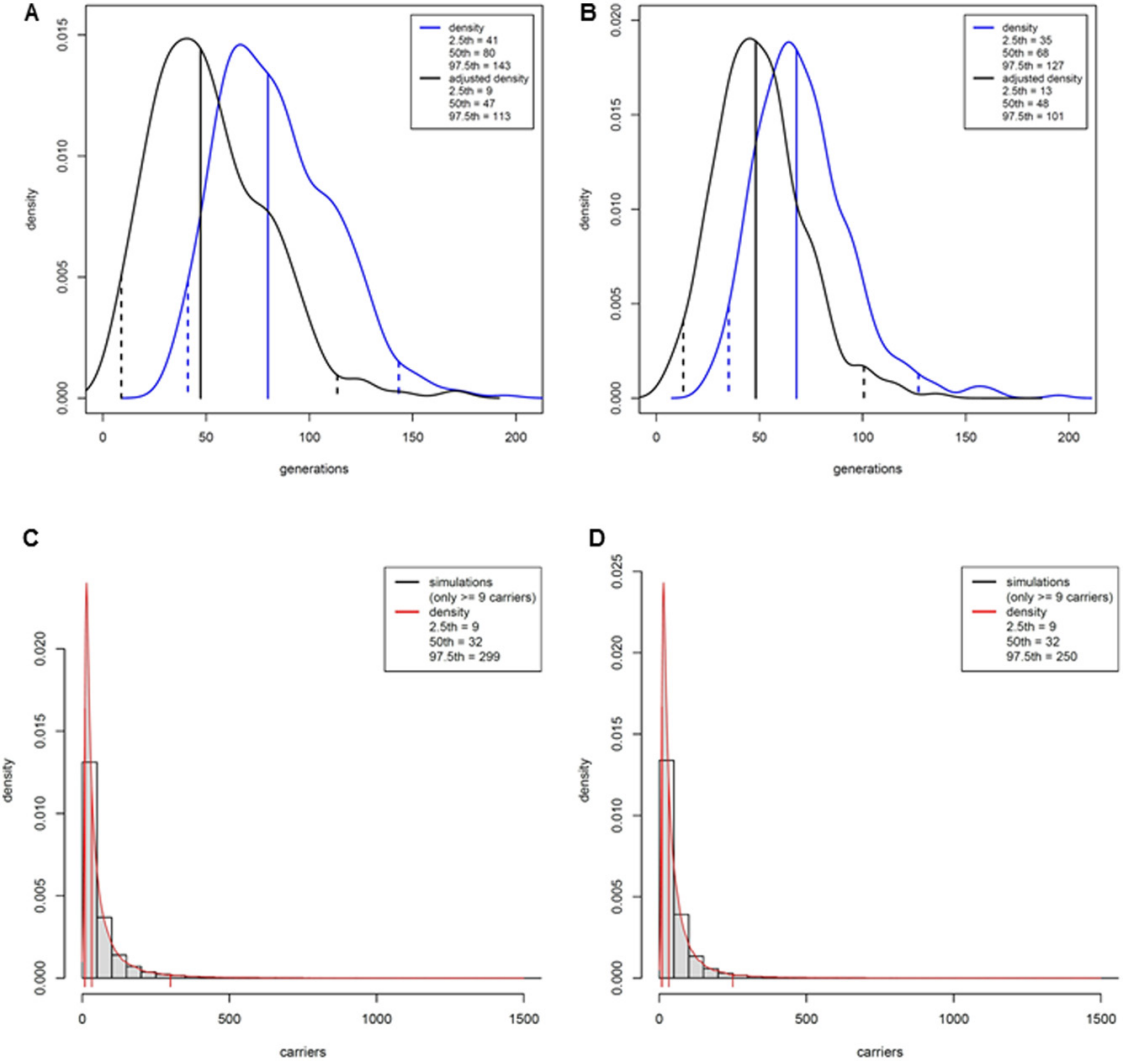
Smoothed distribution of approximate Bayesian computation-simulated time to most recent common ancestor (tMRCA) estimates of the *AIP* c.805_825dup allele, unadjusted (blue curves) and adjusted (black curves) (11), based on HapMap (A) and Rutgers (B) genetic distances. Median, 2.5th and 97.5th percentiles are given in the insert. Distribution of numbers of carriers obtained through forward simulations, calculated based on HapMap-based (C) or Rutgers-based (D) time to most recent common ancestor (tMRCA) estimates randomly sampled from the adjusted distributions shown in A&B. Black bars show density histograms, red curves represent the smoothed distributions. Median, 2.5th and 97.5th percentiles are shown in the insert; the lower bound is conditioned to be nine carriers, the minimum observed number of carriers per generation in our cohort. A full colour version of this figure is available at http://dx.doi.org/10.1530/EJE-17-0293.

We obtained tMRCA estimates of 47 (9–113) generations (median, 95% confidence interval (CI)), based on HapMap genetic distances (22) and 48 (13–101) generations, based on Rutgers map distances (Fig. 3A and B). Assuming a generation time of 25 years (23, 24), these estimates translate to 1175 (225–2825) years and 1200 (325–2525) years respectively.

The estimated current number of carriers per generation – obtained through forward simulations – was 32 (9–299) (median, 95% CI), based on HapMap genetic distances, and 32 (9–250), based on Rutgers map distances (Fig. 3C and D). These results integrate the uncertainty of the tMRCA estimates. Assuming that three 25-year generations co-exist at present, we estimate that 96 (27–897) (median, 95% CI, HapMap-based) c.805_825dup carriers may exist today, of which only 25 have been identified so far.

**Figure 4:**
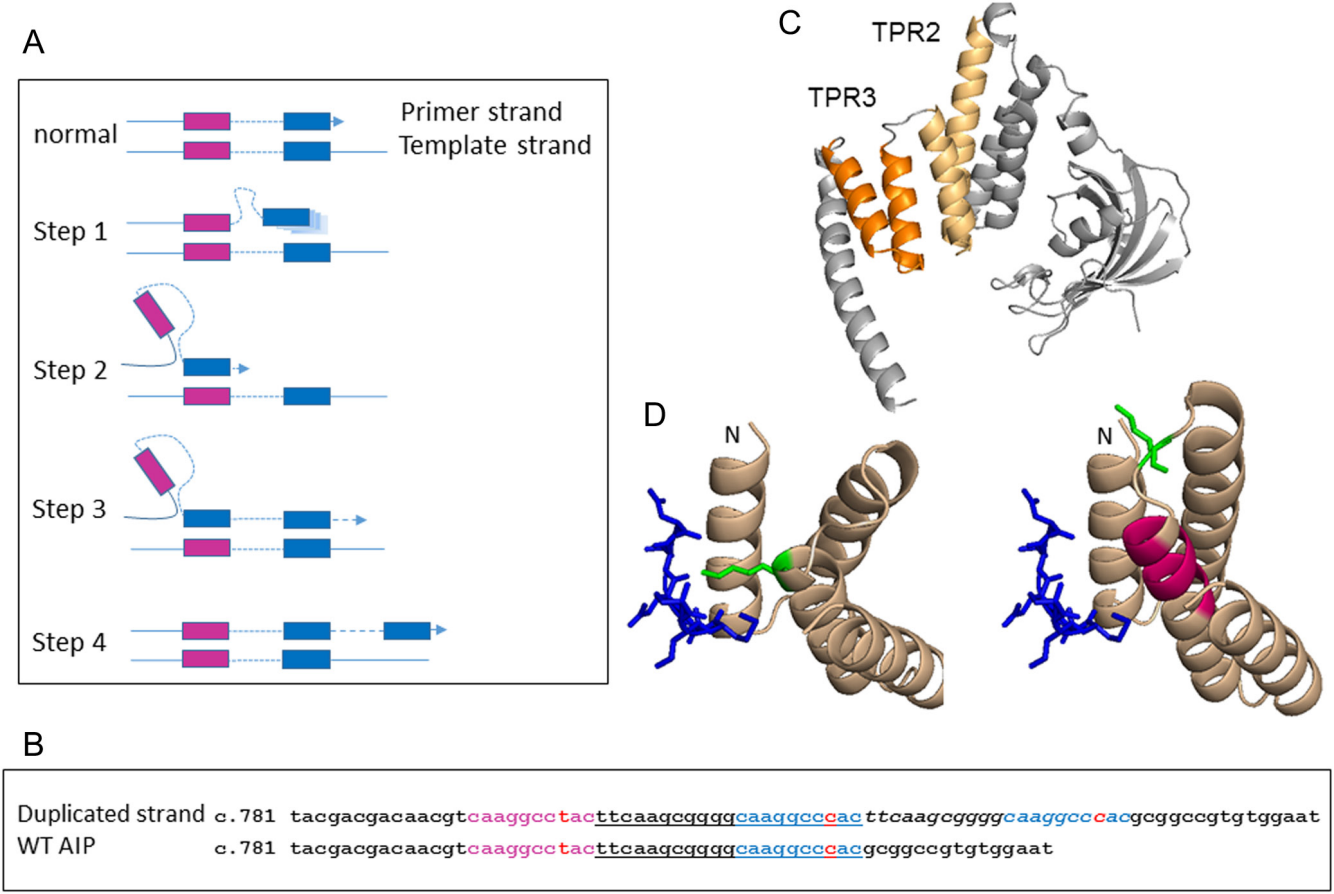
(A) Schematic drawing of the suggested replication slippage mechanism of the c.805_825dup, p.F269_H275dup AIP mutation. The purple and blue colorings match the sequences in (B). During DNA replication, the primer strand containing the newly synthesized second repeat transiently dissociates from the template, slips backward and then re-associates at the first short direct repeat of the template strand, resulting in a misaligned configuration. Continued DNA synthesis will lead to the insertion of intervening sequence and the second direct repeat, resulting in the mutated allele. (B) DNA sequences of WT *AIP* (bottom row) and of the mutated allele (top row), starting at the c.781 base-pair (NM_003977.2). Note the two 9 base-pair long repeats, the 5′ one marked in purple (caaggcctac) and the 3′ one marked in blue (caaggcccac). These are imperfect repeats as the 7th base-pair (in red font) is t in the 5′ repeat and c in the 3′ repeat. The intervening sequence between the two direct repeats is ttcaagcgggg. The underlined sequence is duplicated in the upper sequence, i.e. the mutated allele, in which the first copy of the duplicated sequence is underlined and the second copy of the duplicated sequence is italic. This duplication leads to three copies of the 9 bp repeat: the purple is the 5′ repeat, while the blue is the 3′ repeat which has been duplicated. (C, D and E) Cartoon representation of the AIP protein structure based on the crystal structure of the N and the C-terminal domains of the protein (4, 5). The first and second alpha helices of the WT AIP TPR2 and TPR3 motifs (C) are presented in light orange and dark orange respectively. Details of the wild-type (D) and mutant (E) AIP bound to the SRMEEVD peptide, a fragment of the HSP90 partner protein (shown in blue). The seven amino-acid duplication in the first helix of AIP TPR3 is shown in purple. Residue K266, which in the wild-type AIP interacts with the HSP90 peptide (or with TOMM20 peptide) (5) is displayed as a green stick. A full colour version of this figure is available at http://dx.doi.org/10.1530/EJE-17-0293.

We studied the structure of the DNA sequence around the 21 base-pair tandem duplication allele. We have identified a region in the normal sequence with two short repeated sequences separated by an intervening sequence (Fig. 4A and B), which is typical for a site prone to ‘replication slippage’. Tandem duplications associated with short direct repeats can be explained by the model of ‘replication slippage’ or ‘slipped strand mispairing’ (25, 26). The key feature of this model is that, during replication, the primer strand transiently dissociates from the template, slips backward or forward, and then re-associates at the short direct repeat resulting in a misaligned configuration (Fig. 4A). If the primer strand containing the newly synthesized second (i.e. 3′) repeat dissociates from the template strand and then misaligns at the first (i.e. 5′) direct repeat, continued DNA synthesis will lead to the insertion of one of two direct repeats plus the intervening sequence. In our case, the repeat is imperfect (caaggcctac and caaggcccac) (Fig. 4B), the intervening sequence is ttcaagcgggg and the duplicated region is ttcaagcggggcaaggcccac.

**Figure 5:**
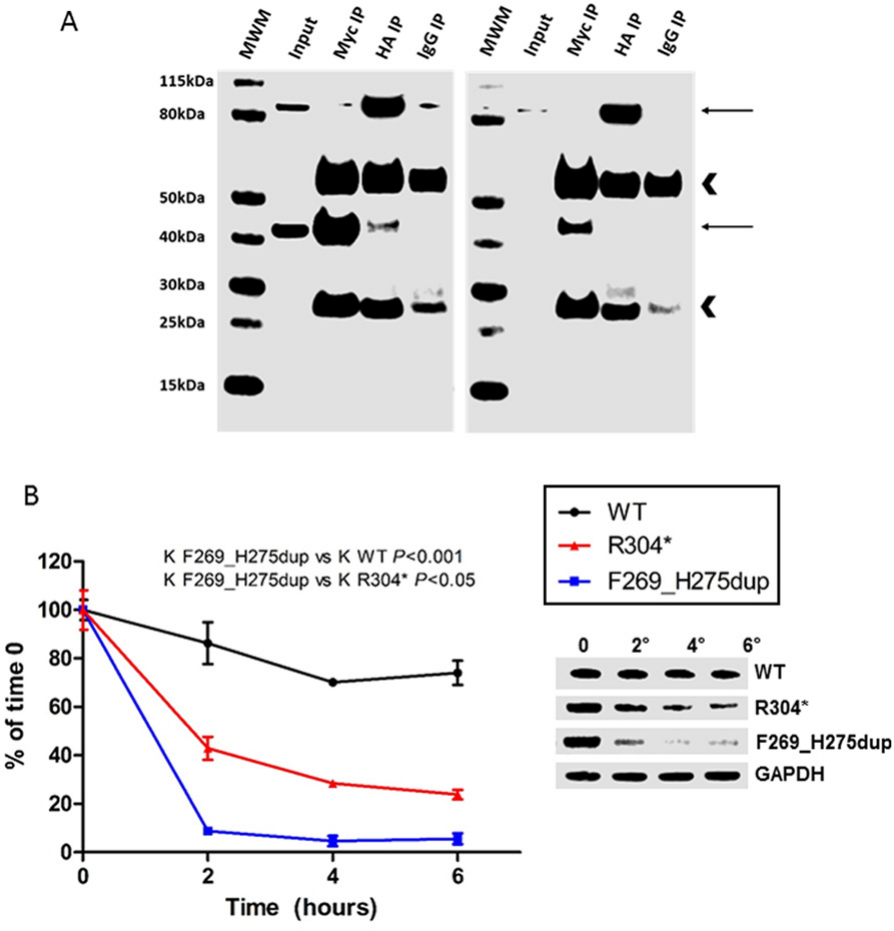
(A) The p.F269_H275dup AIP mutation disrupts the interaction with HSP90. Myc-tagged wild-type (WT) (left panel) or p.F269_H275dup (right panel) AIP were co-transfected with HA-tagged HSP90β in HEK293 cells. Co-immunoprecipitation was performed using anti-Myc or anti-HA mouse antibodies or mouse IgG. Eluates were resolved by denaturing polyacrylamide gel electrophoresis followed by Western blot using anti-Myc and anti-HA antibodies. Positive interaction was demonstrated between WT AIP and HSP90 (a band is also visible in the negative IgG control for HSP90 but not for AIP). No interaction was seen between p.F269_H275dup AIP and HSP90. The steady state levels of mutant AIP were reduced as compared with WT AIP (a band was visible in the Input only after increasing the contrast settings, figure not shown), suggesting that the mutation affects the stability of AIP as well (Fig. 5B). Top arrow: HA-HSP90, bottom arrow: Myc-AIP. Arrowheads: heavy (top) and light (bottom) chains of mouse immunoglobulins. MWM: molecular weight marker. IP: immunoprecipitation. (B) Cycloheximide chase experiment in HEK293 cells overexpressing Myc-tagged WT, p.R304* and p.F269_H275dup AIP. The transfected cells were incubated in the presence of cycloheximide 20 μg/mL for the time indicated. The left panel shows the degradation curves of each protein obtained after plotting the normalized protein levels as percentage of those observed at time 0. The right panel shows representative Western blot images. The degradation speed (K) of the p.F269_H275dup mutant protein was significantly higher than that of WT AIP and that of the truncating mutant p.R304*. A full colour version of this figure is available at http://dx.doi.org/10.1530/EJE-17-0293.

The mutation results in the duplication of seven amino-acids in the third TPR domain of AIP (TPR3). These can adopt a helical conformation, thus adding a 1.9 extra helical turn to the TPR3 alpha helix and increasing its length by 10.5 Å. The TPR3 domain is important for the interaction of AIP with other proteins, in particular, HSP90 and TOMM7 peptides (5). Superposition between the WT C-terminal of the AIP molecule (PDB: 4AIF) and the mutant C-terminal AIP model shows that the seven amino acid duplication may displace K266, which is crucial for HSP90 and possibly other partners binding (Fig. 4C, D and E), thus potentially leading to the disruption of AIP function. The results of a co-immunoprecipitation experiment with mutant AIP and HSP90 were consistent with lack of interaction between the two proteins (Fig. 5A), supporting the results of our *in silico* prediction. As compared with WT AIP, the steady state levels of the p.F269_H275dup protein were markedly reduced, and this led us to hypothesize an effect of the mutation on AIP protein stability as well, consistent with what has already been shown for several other *AIP* mutations (21). Cycloheximide chase experiments showed that the p.F269_H275dup mutation leads to a markedly unstable protein with a degradation constant K of 1.169, significantly higher than that of WT AIP (0.0593; *P* < 0.001) and even that of the truncating mutant p.R304* (0.3147; *P* < 0.05) (Fig. 5B), suggesting shorter protein half-life.

## Discussion

*AIP* mutations are reported in about 20% of FIPA pedigrees (1, 27), but a higher percentage has been observed in homogenous acromegaly families. Over 90 *AIP* mutations have been described to date. The R304 locus seems to be the most prevalent mutational ‘hot spot’, with mutations affecting the c.C910 and c.G911 base-pairs of this CpG site, resulting in R304* and R304Q alleles reported in various countries and unrelated families (28). A few other mutations have also been described as recurring in diverse geographical regions, such as the p.R271W and the p.R81* (3, 29, 30, 31). In this paper, we report nine apparently unrelated pedigrees all carrying the same unique c.805_825dup, p.Phe269_His275dup *AIP* allele. Seven of these pedigrees were not previously reported, and we provide updated information for the other two (3, 9, 13, 20).

When the same allele is identified in different families, the question arises whether this is the result of independent recurrent mutational events in a region of the genome that is more prone to mutations (‘hot spot’, such as CpG sites) (32) or the result of spreading of the disease-associated allele in the population originating from a single founder mutation event. The c.805_825dup, (p.Phe269_His275dup) involves the duplication of 21 base-pairs and probably occurred via the ‘slipped strand mispairing’ mechanism (25, 26, 33) (Fig. 4A). Certain structural features of the genome can predispose a particular region to rearrangement, such as repeated sequences located relatively close to each other, raising the possibility that similar duplications might occur independently. On the other hand, while the patients described here reside in three different areas of the world, all the individuals have ancestral links to the United Kingdom. Furthermore, the steady flow of immigration from Britain to North America in recent centuries and the close proximity of England and France are consistent with an evolutionarily recent common ancestor. Indeed, the result of our haplotype analysis strongly indicates that all patients share a recent common ancestor, the ‘English founder’, who lived approximately 1175 years ago. This estimate has, however, a wide 95% confidence interval of 225–2825 years. The cause of the geographic spreading of this allele is unknown, although mobility in the areas where carriers reside was historically high – particularly since the industrial revolution (34). The penetrance of the pituitary phenotype in patients with mutated *AIP* is known to be low (about 20%) (27). This is confirmed in the largest c.805_825dup-positive pedigree (UK FIPA 1), with an estimated penetrance of 23% (assuming that 50% of the relatives not genotyped would be carriers). Because of this low penetrance, *AIP* mutations do not prevent reproduction in the majority of carriers. Furthermore, even patients who develop pituitary adenomas may maintain normal fertility until the adenoma is large enough to cause hypogonadism. Whether the mutated allele confers some kind of survival advantage is unknown (11).

It is important to note that four of the patients did not have a family history of pituitary adenoma. Following our haplotype analysis, these are unlikely to be *de novo* mutations. These patients were tested for *AIP* mutations because of their clinical characteristics (large adenomas developing at young age, with onset of symptoms ranging between 9 and 20 years), confirming previous reports of relatively high prevalence of *AIP* mutations in apparently sporadic GH-secreting macroadenomas of the youth (27, 35, 36).

The c.805_825dup *AIP* allele is pathogenic, as indicated by the clinical, *in silico* and experimental data. The mutation is predicted to affect AIP structure and function by altering a crucial interaction site between the third TPR domain of AIP (Fig. 4) and one of its most important partners, HSP90 (5), and this has been confirmed experimentally (Fig. 5A). Mutations at protein–protein binding sites are a common cause of disease as they can disrupt the interaction between molecules in cellular protein networks, which is required for correct protein function (37). An alternative pathogenic mechanism by which the c.805_825dup (p.F269_H275dup) affects AIP function is by causing the disruption of AIP protein folding, leading to increased proteasomal degradation, as we have previously shown for numerous *AIP* missense mutations (21). Here, we provided evidence that the p.F269_H275dup mutation markedly affects protein stability, leading to a protein that is significantly less stable compared with the common p.R304* truncating mutant.

In summary, we report the same *AIP* allele occurring in nine apparently unrelated pedigrees from three different (but linked through gene flow) countries and show that all the affected subjects, the majority suffering from young-onset somatotropinomas, share a common ancestral haplotype. The mutation results in the duplication of seven amino-acids in third TPR domain of AIP, leading to the disruption of protein–protein interactions and markedly reduced protein stability.

## Declaration of interest

R S and M K serve on Pfizer Ltd’s advisory board. The other authors declare no conflict of interest.

## Funding

S R was supported by the EC-REA Marie Sklodowska-Curie IEF postdoctoral fellowship (grant 303006/2011). A D was supported by an MRC postdoctoral fellowship (grant MR/K021613/1). D I is supported by a Diabetes UK George Alberti Clinical Research Training Fellowship. M K’s work on familial pituitary adenomas is supported by the Medical Research Council, Barts and the London Charity and Pfizer Ltd.
